# Identification and characterization of PB2 mutations associated with mammalian adaptation of highly pathogenic H5N1 avian influenza viruses

**DOI:** 10.3389/fmicb.2026.1867604

**Published:** 2026-07-29

**Authors:** Simin Cui, Xiaoning Hou, Siyu Pu, Junhao Luo, Haijun Zhu, Peiqing He, Chen Gao, Rongbao Gao, Jun Han

**Affiliations:** National Key Laboratory of Intelligent Tracking and Forecasting for Infectious Diseases National Institute for Viral Disease Control and Prevention, Chinese Center for Disease Control and Prevention, Beijing, China

**Keywords:** H5N1, interspecies transmission, PB2 mutations, PB2 protein, polymerase activity

## Abstract

The highly pathogenic avian influenza virus (HPAIV) subtype H5N1 has been continuously circulating among wild bird populations and domestic poultry. It’s ongoing circulation has led to outbreaks in poultry and U.S. dairy cattle populations, as well as sporadic severe infections in individuals engaged in poultry and dairy farming. These occurrences have raised concerns about the potential evolution of this virus into a pandemic strain. To elucidate the molecular determinants facilitating H5N1 cross-species adaptation and to evaluate its implications for public health, we conducted serials of sequence analysis and specific-site mutations on the viral polymerase subunit PB2 to determine its effect on polymerase activity and viral infectivity. The results showed that three mutations in the PB2 protein (E362G, D441N and M631L) were presented cooperative effects associated with enhanced viral replication in mammalian cells. Compared to the original isolated strain of the 2.3.4.4b clade, A/chicken/NL/FAV-0033/2021, these three mutations were predominantly identified in isolates obtained from cattle and other mammalian hosts between 2021 and 2024. The M631L mutation, identified as the primary determinant of increased polymerase activity in mammalian cells, significantly enhanced the binding affinity of PB2 to ANP32A. The mutation E362G and D441N did not increased polymerase activity and viral replication significantly but enhanced binding affinity of PB2 to ANP32A. The combined mutations with E362G, D441N and M631L resulted in a significantly increased polymerase activity and viral replication in H5N1 virus, and significantly elevated viral loads and aggravated pulmonary pathology in lungs of mice with H5N1 infection. These findings indicate that the PB2-M631L mutation constitutes a crucial molecular marker for the adaptation of H5N1 to mammalian hosts, whereas the E362G and D441N mutations likely function as supportive modulatory factors that optimize this host-adaptation process.

## 1 Introduction

Since the initial identification of the highly pathogenic avian influenza virus H5N1 (A/Goose/Guangdong/1/1996, GsGD) in China in 1996, the GsGD lineage has disseminated globally via migratory birds. This virus is capable of infecting poultry, wild birds and a range of mammalian species, and has occasionally been reported to infect humans, posing a persistent threat to global public health (Welkers et al., 2019). Since the initial documented instance of cross-species infection in humans in Hong Kong in 1997, over 900 cases of human infection with a up to 50% fatality have been reported across 23 countries worldwide as of 2025, representing a significant zoonotic disease risk (Welkers et al., 2019; Lee et al., 2015; Lewis et al., 2021; Webby and Webster, 2003; Imai et al., 2013). The 2.3.4.4b subclade HPAIV H5N1 viruses circulating predominantly in 2021 had the broadest spectrum of mammalian infections within the H5 GsGD evolutionary lineage, and were reported to infect more than 50 species of mammals including terrestrial carnivores (such as foxes, otters, minks, dogs, and cats) and marine mammals (such as seals, sea lions, elephant seals, and dolphins) (Leguia et al., 2023; Uhart et al., 2024; Burrough et al., 2024; Moreno et al., 2023). And the virus has caused widespread outbreaks in U.S. dairy cattle, along with isolated human cases Since March 2024, and raised concerns again about its increased risk on enhanced transmissibility and potential pandemic (Caliendo et al., 2022; Mostafa et al., 2024; Caserta et al., 2024; Nelli et al., 2024; Eisfeld et al., 2024)_._

The primary factors facilitating the cross-species transmission of avian influenza viruses from birds to mammals encompass alterations in the hemagglutinin (HA) protein receptor binding specificity, mutations in the viral polymerase complex components (PB2, PB1, and PA) and mechanisms of immune evasion (De Graaf and Fouchier, 2014; Moncorge et al., 2010). Previous studies have identified several critical mammalian-adaptive substitutions in PB2 such as E627K, D701N, Q591K and M631L, which can help virus to overcome host restrictions by enhancing polymerase stability and recruiting host cofactors through facilitating viral RNA synthesis and increasing viral replication (Salomon et al., 2006; Fouchier et al., 2004; Hatta et al., 2001; Le et al., 2003; Shinya et al., 2004; Zhou et al., 2011; Restori et al., 2024; Bussey et al., 2010). Since the widespread dissemination of the Eurasian H5N1 2.3.4.4b clade throughout Europe in 2020, the virus subsequently crossed the Atlantic Ocean to North America by late 2021, and a novel reassortant genotype B3.13 emerged during outbreak of America dairy cattle in 2024 (Jahid and Nolting, 2025). Phenotypically, the B3.13 virus was demonstrated to have cross species transmissibility via direct contact, and presented a broad infectivity across pigs, rats, and ferrets. The virus presented a strong replication capability in human tracheal epithelial cells although maintaining a preferential binding affinity for avian-like *α*-2,3-linked sialic acid receptors (Fabrizio et al., 2025). Identification of novel characteristics contributing to mammalian adaptation would be important for understanding the potential risk of the virus on public health although the PB2 protein of B3.13 virus does not contain the previously identified classical mammalian adaptation mutations.

In this study, we analyzed the evolutionary dynamics of PB2 sequences from H5N1 outbreaks in U.S. dairy cattle, and identified key adaptive mutations in the PB2 protein enhancing polymerase activity and viral replication capability as well as pathogenicity in mice. These finding would be helpful to understand the viral cross species transmission and its potential risk on public health.

## 2 Materials and methods

### 2.1 Virus sequence collection and evolutionary analysis

A dataset comprising 812 PB2 sequences from H5N1 clade 2.3.4.4b isolates collected between 2021 and 2024 was obtained and assembled from the GISAID database. Those viruses in the dataset were from a diverse array of viral genotype A1, A2, B1.3, B3.2, Minor01, B3.6, B3.7, B3.9 and B3.13, and derived from various host species encompassing avian reservoirs as well as mammals such as skunks, red foxes, harbor seals, cats, cattle and humans. Multiple sequence alignments were conducted using MEGA version 7.0, and phylogenetic relationships were inferred employing the Maximum Likelihood (ML) method. To quantify sequence variability, we calculated site-specific Shannon entropy as a measure of amino acid conservation. The host and temporal sequence characteristics of the PB2 protein mutation sites were determined by the Nextstrain platform.^1^

### 2.2 Protein structure prediction and molecular docking

The three-dimensional structure of the PB2 protein was predicted by AlphaFold3^2^ and Swiss-Model^3^ online platforms. The mutation site E362G, D441N or M631L in PB2 protein are located within distinct structural and functional domains of the protein. The structures were visualized using PyMOL software (version 3.0.3). ANP32A and ANP32B are critical host factors for sustaining the activity of the influenza virus polymerase. Adaptation of the PB2 protein to mammalian-type ANP32 via specific mutations enables the virus to circumvent host restrictions by enhancing its binding affinity to mammalian ANP32, thereby restoring polymerase function and facilitating cross-species replication (Long et al., 2016; Mistry et al., 2020). A molecular docking analysis was conducted to examine the interaction between PB2 and ANP32A (PDB ID: 4XOS), and to compare the binding affinities and interface conformational alterations between the wild-type and PB2 mutants utilizing the HDOCK docking server.^4^

### 2.3 Construction of viral mutants

PB2 mutant H5N1 viruses were rescued using reverse genetics techniques using an ukaryotic expression vector pHW2000 containing the human RNA polymerase I promoter alongside the human cytomegalovirus RNA polymerase II promoter (Hu et al., 2015). The genomic sequence of A/goose/Colorado/23-038138-001/2023 (H5N1) virus sequence (EPI_ISL_19593741) obtained from GISAID as a reference. Complementary DNA (cDNA) corresponding to each of the eight viral gene segments was individually cloned into pHW2000 to construct recombinant plasmid pHW2000-HA, pHW2000-NA, pHW2000-NP, pHW2000-NS, pHW2000-PB1, pHW2000-PA, pHW2000-M and pHW2000-PB2 (containing four mutants designated as pHW2000-PB2-E362G, pHW2000-PB2-D441N, pHW2000-PB2-M631L and pHW2000-PB2-E362G + D441N + M631L). To facilitate the handling of the rescued virus in a Biosafety Level 2 (BSL-2) laboratory environment, the multibasic cleavage site sequence PLREKRRKR/G in the hemagglutinin (HA) gene was changed in the naturally occurring sequence PQRE---TR/G. For viral rescue, human embryonic kidney 293 T cells were seeded at a density of 1.5 × 10^6^ cells per well in 6-well culture plates, and were incubated to reach 80 to 90% confluency at 37 °C in a humidified atmosphere containing 5% CO_2_ for 12 to 24 h. Eight recombinant plasmids with the 8 segments of H5N1 virus were co-transfected into the 293 T cells at a concentration of 500 ng each using Lipofectamine™ 3000 reagent following the manufacturer’s instructions. Six hours after transfection, the culture medium was replaced with a suspension of Opti-MEM with TPCK-trypsin at 2 μg/mL. After a 48-h incubation, 100 μL supernatant was collected to inoculate into 9- to 10-day-old embryonated chicken eggs to promote viral amplification. Following a two-hour incubation period, collect the blastocoel fluid and conduct a red blood cell agglutination assay utilizing turkey red blood cells to assess viral activity. Virus samples that produced positive results in the experiment were considered successfully isolated. These isolated virus samples were then stored at −80 °C for subsequent use.

### 2.4 Determination of viral tissue culture infectious dose 50% (TCID₅₀)

MDCK cells were seeded in 96-well plates at a density of 2.5 × 104 cells/well and incubated overnight. Serial semi-logarithmic dilutions of viral stocks or samples were prepared in viral maintenance medium (DMEM supplemented with 0.2% BSA and 2 μg/mL TPCK-treated trypsin), and 100 μL of each dilution was inoculated onto the monolayers. After adsorption for 1 h at 37 °C, the inoculum was removed, and cells were washed and replenished with 200 μL of fresh maintenance medium. Plates were incubated for 72 h and monitored daily for cytopathic effects (CPE). Endpoint titers were determined by assessing hemagglutination (HA) activity in the supernatants, and the 50% tissue culture infectious dose (TCID50) was calculated using the Reed–Muench method.

### 2.5 Measurement of influenza virus polymerase activity

To assess the functional impact of PB2 amino acid substitutions, polymerase activity was evaluated using a luciferase-based viral ribonucleoprotein (RNP) reconstitution assay (Eisfeld et al., 2024). HEK293T and DF-1 cells were seeded in 24-well plates (3 × 105 cells/well) and incubated overnight. Recombinant RNP complexes were reconstituted by co-transfecting cells using Lipofectamine™ 3000 with a plasmid mixture containing 200 ng each of pHW2000-PB1, pHW2000-PA, pHW2000-NP, the vRNA-like reporter (pPOL I-Luci-NP), and the specified pHW2000-PB2 variant (WT, E362G, D441N, M631L, or the triple-substitution variant). pRL-TK (Renilla luciferase) in 20 ng was co-transfected in each reaction as an internal control for transfection efficiency. At 48 h post-transfection, polymerase activity was quantified using the Dual- Glo^®^ Assay System on a Synergy 2 microplate reader (BioTek). Firefly luciferase luminescence was normalized to Renilla activity, and data are reported as the mean ± s.d. derived from three independent biological replicates (*n* = 3).

### 2.6 Western blotting

To assess the expression level of the PB2 protein, plasmids pHW2000-PB1, pHW2000-PA, pHW2000-NP, and plasmids encoding various PB2 mutants (or PB2 with a Flag tag) were co-transfected into 293 T cells using Lipofectamine™ 3000 reagent. At 24 h post-transfection, HEK293T cells co-transfected with plasmids encoding the viral RNP complex (PB1, PA, NP) and the indicated wild-type or mutant PB2 constructs (including Flag-tagged variants) were lysed in ice-cold RIPA buffer, and total protein concentration was determined through BCA protein assay kit. Equal protein loads were resolved by SDS-PAGE and transferred to PVDF membranes. Membranes were blocked with 10% nonfat milk and probed with rabbit anti-PB2 (1:2,000, GeneTex) or anti-*β*-actin (1:5,000, Proteintech), followed by HRP-conjugated goat anti-rabbit IgG (1:5,000, Abcam). Immunoreactive bands were visualized using enhanced chemiluminescence (ECL).

### 2.7 Subcellular localization of PB2 protein

Influenza virus transcription and replication occur in the nucleus of infected host cells. As an essential component of the viral RNA-dependent RNA polymerase complex (PB1–PB2–PA), PB2 must be successfully imported into the nucleus to perform its function. Mutations that cause PB2 to accumulate in the cytoplasm may disrupt its nuclear import signals or interfere with its interaction with host nuclear import factors such as importin-*α*, thereby diminishing viral replication efficiency in mammalian cells (Resa-Infante and Gabriel, 2013). To analyze subcellular distribution, HEK293T cells were seeded onto poly-L-lysine–coated 24-well plates (3 × 10^5^ cells/well) to culture overnight, and then were co-transfected with plasmids encoding the viral polymerase complex (PB1, PA, NP) and either wild-type or mutant PB2 (E362G, D441N, M631L) via Lipofectamine™ 3000. At 24 h post-transfection, cells were fixed with 4% paraformaldehyde, permeabilized with 0.5% Triton X-100, blocked with 3% BSA, and were immunostained with rabbit anti-PB2 (1:500; GeneTex) followed by Alexa Fluor 488–conjugated goat anti-rabbit IgG (1:400; Abcam). Nuclei were counterstained with DAPI (Invitrogen). The cellular localization of the stains was visualized using confocal microscopy (Nikon Instruments, Japan).

### 2.8 Co-immunoprecipitation, co-IP

To examine the impact of PB2 amino acid substitutions on the recruitment of the host factor ANP32A, HEK293T cells were co-transfected using Lipofectamine™ 3000 with plasmids expressing Flag-tagged PB2 variants (WT or indicated substitutions) and ANP32A. After 24 h, whole-cell lysates were prepared in ice-cold RIPA buffer. Protein complexes were immunoprecipitated using Anti-Flag magnetic beads (LABLEAD, PFM025) at 4 °C for 2 h. The immunoprecipitates were eluted, separated by SDS–PAGE, and analyzed by Western blotting using anti-PB2 (GeneTex, 637893) and anti-ANP32A (GeneTex, 115933) antibodies. Relative binding efficiency was assessed by comparing the band intensities of ANP32A co-eluted with each PB2 variant.

### 2.9 Viral replication efficiency and transcriptional analysis

This study employed reverse genetics technology to generate recombinant H5N1 virus strains harboring distinct PB2 mutations, in order to compare their replication efficiency and transcriptional activity across various cell lines. Subsequently, the MDBK, A549, DF-1 and PK-15 cell lines were infected at a multiplicity of infection (MOI) of 0.1. Cell culture supernatants were collected at 24, 48, and 72 h post-infection, and viral titers were quantified using the 50% tissue culture infectious dose (TCID50) assay.

Total RNA was extracted from infected A549 cells at 8 h post-infection (hpi) using the RNeasy Mini Kit (Qiagen) in accordance with the manufacturer’s protocol. To rigorously distinguish between negative-sense genomic vRNA and positive-sense viral mRNA species, reverse transcription (RT) was conducted as a separate first step employing two strand-specific priming strategies. Specifically, genomic negative-sense vRNA was selectively reverse-transcribed using the Uni-12 primer (5′-AGCAAAAGCAGG-3′), which complementarily targets the highly conserved 12-nucleotide sequence at the 3′ end of the viral genomic segments. Conversely, viral mRNA was selectively targeted and reverse-transcribed using an oligo(dT) primer that complementarily binds only to the poly(A) tails of viral transcripts, thereby preventing the reverse transcription of non-polyadenylated genomic vRNA. Both RT reactions were performed using standard reverse transcriptase (Beijing Xinke Open Source) to generate strand-specific cDNA.

Specific primers and probes targeting the nucleoprotein (NP) and matrix protein 1 (M1) gene segments of the influenza virus were independently designed in our laboratory. The sequences employed in this study are specified as follows:

**Table:** 

Target gene	Primer/probe	Sequence (5′–3′)
NP	NP-F	GGTAGATAATCACTCACTGA
NP	NP-R	GCACATCTGTATGTAGAATC
NP	NP-Probe	TCCGCCAACCATTCTTCCAACA
M1	M1-F	CTCACAGACAGATAGCTAC
M1	M1-R	CTAGGATGAGTTCCAATGG
M1	M1-Probe	CACCATCTGCCTAGCCTGACT

These primers and probes were synthesized by Beijing Xinke Open Source Gene Technology Co., Ltd. and have been validated for their efficacy. To evaluate the assay parameters, plasmid standards containing the target NP or M1 fragments were prepared in a 10-fold dilution series (dilution range 10^−1^ to 10^5^), and a standard curve was plotted accordingly. The amplification efficiency (E) for both NP and M1 detection was calculated based on the slope of the standard curve. The R^2^ result was 0.996, and the linear relationship was excellent (coefficient of determination *R*^2^ > 0.99), with typical Ct values ranging from 14 to 1.

**Table:** 

Target gene	Primer/probe	Sequence (5′–3′)
*β*-actin	Forward primer	TGCGTGACATCAAGGAGAAG
*β*-actin	Reverse primer	TGCCAGGGTACATTGTGGTA
*β*-actin	Probe	TTCCTGGGCATGGAGTCCTGTGGC

To account for variations in cell number and RNA input, viral copy numbers were normalized to the levels of the cellular housekeeping gene *β*-actin. *β*-actin expression was quantified using the following oligonucleotide sequences:

The data obtained were normalized and expressed as the number of viral copies per 10^5^ β-actin transcripts. To ensure reliability and reproducibility, all assays were performed in triplicate.

### 2.10 Ethics statement and animals

The animal study design, execution, and reporting were conducted in strict accordance with the ARRIVE guidelines 2.0 (Animal Research: Reporting of *In Vivo* Experiments). All animal experiments were approved by the Institutional Animal Care and Use Committee (IACUC) of the National Institute for Viral Diseases Control and Prevention of the China CDC (approval number: 20221107112) and were conducted in strict accordance with the guidelines for the care and use of laboratory animals. Six-week-old specific-pathogen-free (SPF) female C57BL/6 J mice were purchased from (Beijing Vital River Laboratory Animal Technology Co., Ltd.). The mice were housed in isolators within a Biosafety Level 3 (BSL-3) containment facility with ad libitum access to food and water. All inoculated mice were included in the analysis. No exclusion criteria were applied.

To assess pathogenicity, mice were lightly anesthetized with isoflurane via inhalation and inoculated intranasally with 50 μL of viral suspension containing 10^3^ TCID₅₀ of the respective rescued viruses (WT, E362G, D441N, M631L, or the triple-mutant). A control group was mock-inoculated with an equal volume of phosphate-buffered saline (PBS). Body weight and survival were monitored daily for 14 days post-infection (dpi). Mice that lost >25% of their initial body weight were considered to have reached the humane endpoint and were euthanized by CO₂ inhalation followed by cervical dislocation, in strict accordance with the AVMA Guidelines for the Euthanasia of Animals. On day 9 post-infection, mice from the groups showing significant weight loss were humanely euthanized, and lung tissue was harvested for subsequent analysis. Mice in the remaining groups were monitored for weight changes until the end of the experiment.

### 2.11 Histopathological and immunohistochemical examination

Lungs were fixed in 10% neutral-buffered formalin for 48 h, embedded in paraffin, and sectioned at a thickness of 4–5 μm. Sections were deparaffinized in xylene and rehydrated through a graded ethanol series. Hematoxylin and eosin (H&E) staining was performed according to standard protocols to evaluate pathological changes. Histopathological lesions were examined and imaged using a light microscope (Olympus, Tokyo, Japan). For IHC analysis, paraffin-embedded sections were deparaffinized and rehydrated as described above. Antigen retrieval was performed by boiling sections in citrate buffer (pH 6.0) for 15 min. Endogenous peroxidase activity was blocked with 3% hydrogen peroxide for 10 min, followed by blocking with 5% BSA for 30 min at room temperature to prevent non-specific binding. Sections were then incubated overnight at 4 °C with a primary antibody against the Influenza A Virus Nucleoprotein (NP). After washing with PBST, sections were incubated with a HRP-conjugated secondary antibody for 1 h at room temperature. The reaction was visualized using 3,3′-diaminobenzidine (DAB) substrate, and nuclei were counterstained with hematoxylin. Images were captured using a light microscope.

### 2.12 Quantitative and statistical analysis

All statistical analyses and graph generation were performed using GraphPad Prism software (version 10). For comparisons involving multiple experimental groups, statistical significance was evaluated using One-way or Two-way analysis of variance (ANOVA). To strictly control the family-wise error rate during multiple testing, Dunnett’s *post-hoc* test was applied. Calculated multiple-comparison values were reported as adjusted *p*-values. In accordance with the reviewer’s recommendation for optimal transparency, significance asterisks were replaced, and exact adjusted *p*-values (or specific intervals, such as *p* < 0.001) were directly annotated on the charts. Corresponding 95% confidence intervals (95% CI) for key datasets are further detailed within the Results text. Differences were considered statistically significant when the adjusted *p* < 0.05.

## 3 Results

### 3.1 PB2 genetic analysis and protein mutation site screening

**Figure 1:**
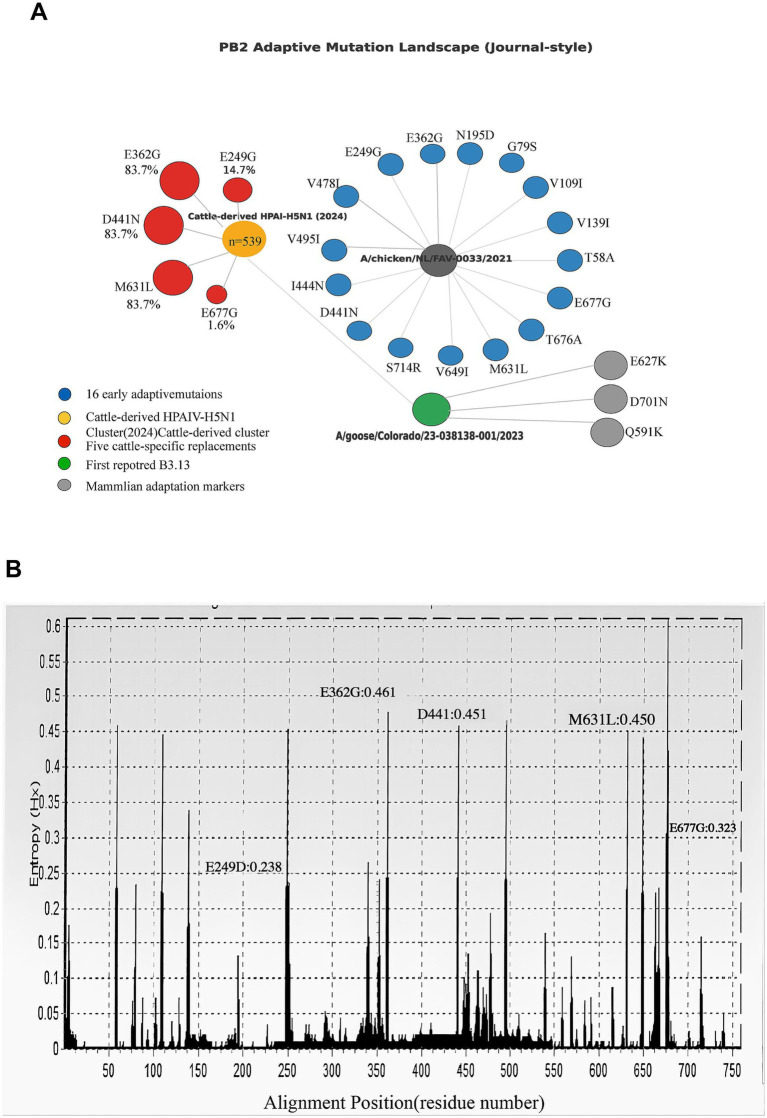
Phylogenetic profiles and site-specific variability of PB2 mutations. Amino acid variation map of the PB2 protein from U.S. H5N1 (clade 2.3.4.4b) viruses collected between 2021 and 2024. Blue circles indicate 16 early adaptive amino acid mutations identified in the PB2 protein during this period. Orange circles represent representative sequences obtained from cattle, humans, poultry and non-human mammals in the United States, collected between March and July 2024 (*n* = 539). Gray circles denote the positions of canonical mammalian-adaptive mutations (PB2-E627K, D701N, Q591K) and are provided for reference purposes. Green circle denote early representative sequences of the B3.13 lineage, specifically A/goose/Colorado/23-038138-001/2023. Red circles denote five novel amino acid substitutions (E249G, E362G, D441N, M631L, E677G) identified in the highly pathogenic avian influenza (HPAI) H5N1 B3.13 virus sequences isolated from U.S. dairy cattle. The mutations PB2-E362G, PB2-D441N and PB2-M631L each reached a prevalence of 83.7%, while the PB2-E249G mutation was detected at a frequency of 14.7% and the PB2-E677G mutation at 1.6%. (B) Shannon entropy analysis of 812 PB2 sequences. The *x*-axis represents amino acid positions and the *y*-axis indicates entropy measured in bits. Elevated Shannon entropy values correspond to increased amino acid variability and reduced evolutionary conservation at specific positions, whereas lower values signify greater conservation. Sites PB2-E362G, D441N, and M631L exhibit elevated entropy values (0.461, 0.451, and 0.450, respectively).

The study employed the sequence of the first 2.3.4.4b clade H5N1 virus sequence (A/chicken/NL/FAV-0033/2021) as a reference to perform a systematic analysis of 812 PB2 protein sequences from highly pathogenic avian influenza (HPAI) H5N1 viruses collected in the United States between 2021 and 2024. These sequences encompassing a broad spectrum of viral genotypes (e.g., A1, A2, B1.3, B3.2, Minor01, B3.6, B3.7, B3.9, and B3.13) were obtained from both avian and mammalian hosts including skunks, red foxes, harbor seals, humans, cats, and cattle. Analysis of the North American clade 2.3.4.4b lineage demonstrated that a set of 16 PB2 mutations characteristic of the B3.13 genotype has been consistently retained since 2022 (Figure 1, blue circles). Compared with representative avian-derived sequences from the global H5N1 peak in 2014–2015 (*n* = 773), all 17 mutations of these sites represent novel amino acid substitutions unique to the B3.13 genotype. By comparing isolates collected during the outbreak period (March–July 2024; *n* = 539) with the earliest B3.13 reference (A/goose/Colorado/23-038138-001/2023)A/goose/Colorado/23-038138-001/2023, we identified five acquired substitutions—E249G, E362G, D441N, M631L and E677G—that emerged subsequent to the virus’s establishment in cattle populations (Figure 1A, red circles).

Among these, E362G, D441N, and M631L were predominant, reaching a collective frequency of 83.7%, whereas E249G (14.7%) and E677G (1.6%) remained sub-dominant. To further assess the conservation of these five mutations, we calculated entropy values for PB2 protein sequences of HPAI H5N1 viruses collected between 2021 and 2024 using the Entropy tool. The entropy scores for PB2-E249G, PB2-E362G, PB2-D441N, PB2-M631L, and PB2-E677G were 0.238, 0.461, 0.451, 0.450, and 0.323, respectively, (Figure 1B). In protein evolution study, increased entropy indicates greater amino acid variety and hence reduced conservation (Steel et al., 2009). Based on the combined frequency and entropy metrics, E249G and E677G were excluded from subsequent analyses in this study.

### 3.2 Evolutionary patterns of PB2 mutations across hosts and time series

**Figure 2:**
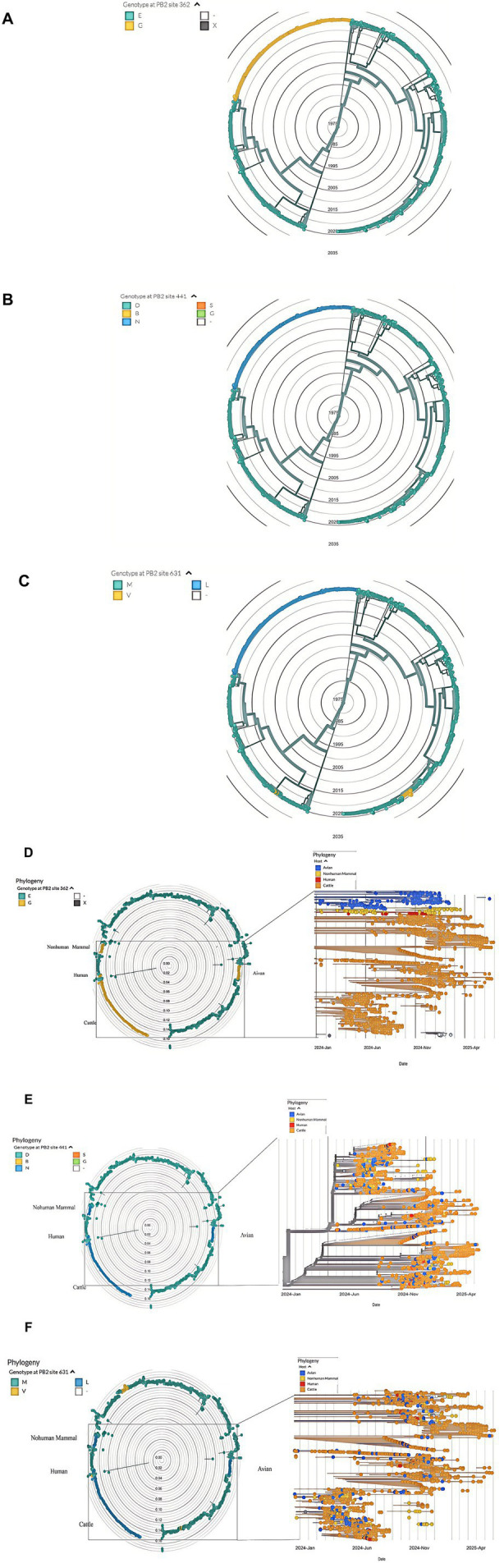
Temporal and host distribution of key PB2 mutations. Annual frequency patterns of the PB2 substitutions at amino acid residue 362 **(A)**, 441 **(B)** and 631 **(C)**. The different colors represent distinct amino acids at the positions of the residues, as indicated by the squares located in the top left corner of each panel. **(D–F)** The distribution of PB2-E362G **(D)**, PB2-D441N **(E)** and PB2-M631L **(F)** among avian species, cattle, humans, or other mammalian hosts in U.S. HPAI H5N1 detections since March 2024. The scale bars denote genetic divergence, quantified as nucleotide substitutions per site in relation to the root of the phylogenetic tree. Host categories are indicated by colors: blue for avian species, yellow for non-human mammals, red for humans and orange for cattle. The enlarged panels highlight the distribution of the B3.13 genotype across different host species between March 2024 and April 2025.

We acquired and curated PB2 protein sequences of H5N1 viruses gathered from 2021 to 2024, resulting in over 4,000 entries from avian, human, and bovine hosts, utilizing a dataset sourced from the Nextstrain platform. Temporal frequency analyses revealed that all three mutations E362G, D441N, and M631L presented extremely low levels (<1% overall) with most avian-derived strains retaining the wild-type residues prior to 2021, and the frequencies of the mutations had significantly risen to 94.96%for PB2-E362G, 94.96% for PB2-D441N, and 97.83% for PB2-M631L By 2024 while the mutations were predominantly detected in cattle and other mammalian hosts this time. Furthermore, from 2024 onwards, the B3.13 lineage became predominant, and the triple-mutant haplotype (E362G/D441N/M631L) became the dominant signature of viruses from cattle, humans, and other non-human mammals, while remaining sporadic in avian strains (Figure 2).

### 3.3 PB2 protein structural modeling and host factor binding analysis

**Figure 3:**
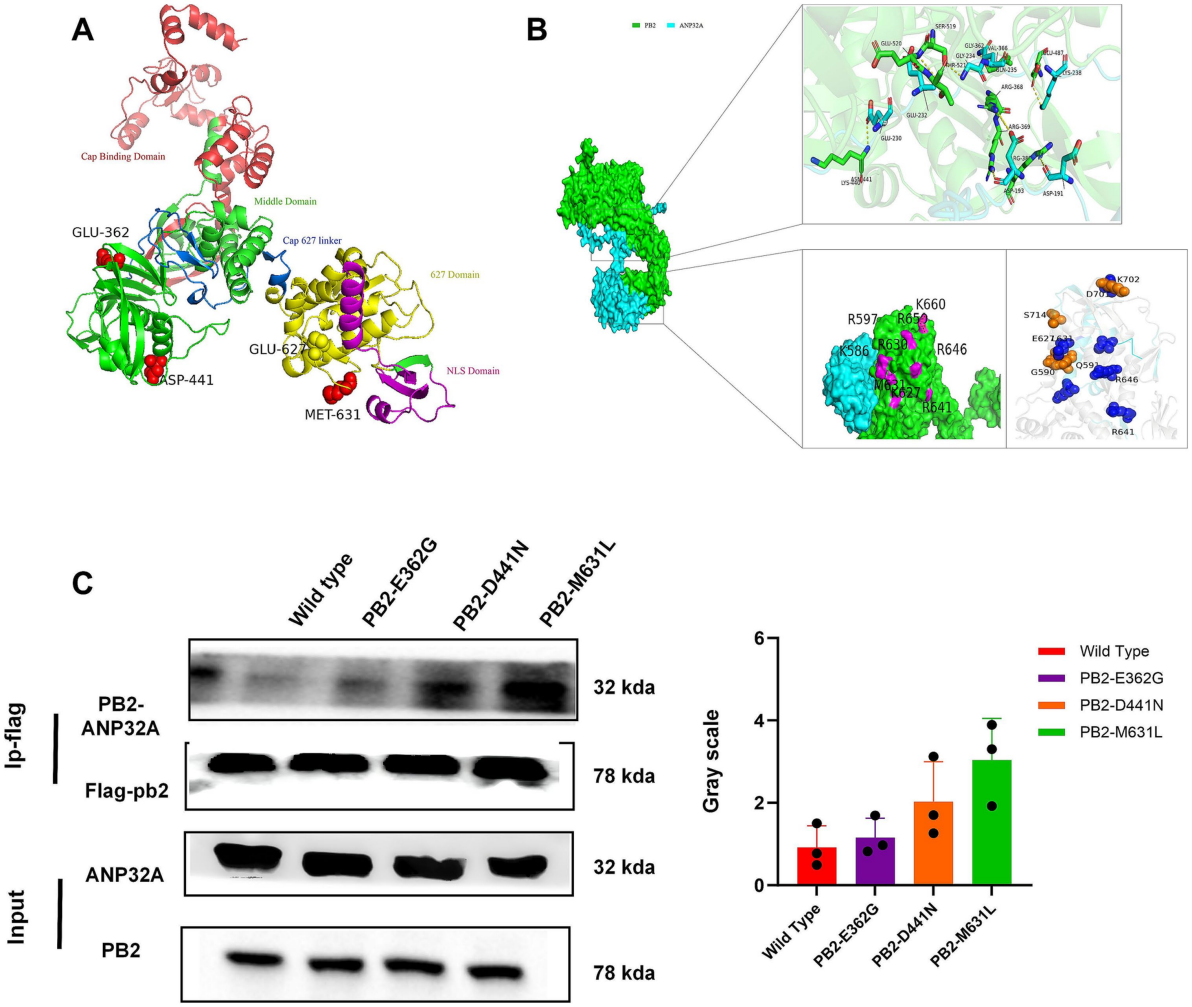
Structural modeling and PB2–ANP32A interaction analysis. **(A)** The domain architecture of PB2, as predicted by AlphaFold3, encompasses the cap-binding domain (CBD), the mid domain, the 627 domain and the nuclear localization signal (NLS). The mutation sites E362, D441, and M631 are indicated. **(B)** Docking of PB2 to the host protein ANP32A (PDB ID: 4XOS) was conducted using HDOCK, resulting in a predicted docking score of −264.36 kcal/mol. **(C)** Co-IP analysis of PB2–ANP32A interaction. Quantified band intensities (right) are presented as the mean ± standard deviation from three independent experiments. Statistical significance was determined by One-way (or Two-way) ANOVA followed by Dunnett’s *post-hoc* test for multiple comparisons. Error bars represent the mean ± standard deviation (SD) from three independent biological replicates. In accordance with the reviewer’s recommendation for optimal transparency, significance asterisks were replaced, and exact adjusted *p*-values (or specific intervals, such as *p* < 0.001) were directly annotated on the charts. Differences were considered statistically significant when the adjusted *p*-value was less than 0.05.

The PB2 subunit of influenza A virus RNA polymerase consists of multiple functional domains including a cap-binding domain (approximately amino acids 100–250), a middle domain (approximately residues 320–480), a cap-627 linker, a 627 domain named for host-range determinant residue 627 and a C-terminal nuclear localization signal (NLS) domain (Figure 3A). PB2-E362G is situated within a helical segment of the middle domain, adjacent to the transcription initiation interface. The substitution of an acidic residue with a small neutral glycine at this position may modulate the local physicochemical microenvironment. PB2-D441N resides in a separate *α*-helix downstream of residue 362, also within the middle domain but spatially proximal to the 627 domain. The PB2-M631L substitution is located in a loop of the flexible 627–631 region and may subtly alter the local surface or conformational dynamics of this region, thereby influencing interactions with host cofactors such as ANP32A. Root-mean-square deviation (RMSD) was used to quantify the average positional displacement of corresponding atoms between structural models, with RMSD values below 1.0 Å generally indicating a high degree of atomic-level similarity. Comparison between the wild-type and mutant PB2 structures yielded a backbone RMSD of 0.007 Å across the mutants, indicating that the overall three-dimensional architecture of the mutant proteins remains largely consistent with that of the wild type (Figure 3A).

Molecular docking simulations indicated that the C-terminal region of ANP32A and the middle domain of PB2 (residues 307–534) are critical for mediating the interaction between PB2 and ANP32A (Table 1). The residue GLN235 and Glu230 on ANP32A form a stable interface interaction network with the residue VAL366, ARG368, and LYS440 on PB2 (Figure 3B). The 362 residue is in close proximity to key aromatic amino acids (Phe363 and Phe404), the presence of 362G may slightly increase the distance between these aromatic rings in the predicted structure of bovine PB2, and affecting the PB2 cap-binding activity. The PB2-M631L mutation resides within the PB2 flexible auxiliary region (627–631 loop), with the side chain of this residue oriented towards the protein surface adjacent to key residues K589, R630, and R641. Together, these residues form a ‘positive charge ridge’ that interacts with the ANP32A LRR domain, establishing a dynamic charge-complementary network with the acid-rich tail within the intrinsic disordered domain (IDD) of the ANP32A protein.

**Table 1:** PB2 protein and ANP32A molecular docking predicted binding site.

Structure1-chain	Structure1-residue	Distance	Structure2-chain	Structure2-residue
A:PB2	ARG 46	3.78	B:ANP32A	GLU 10
A:PB2	LYS 41	2.23	B:ANP32A	GLU 70
A:PB2	ARG 38	3.67	B:ANP32A	ASP 73
A:PB2	LYS 140	3.35	B:ANP32A	GLU 177
A:PB2	ARG 216	2.29	B:ANP32A	GLU 181
A:PB2	THR 215	3.37	B:ANP32A	GLN 184
A:PB2	ARG 144	3.67	B:ANP32A	GLN 184
A:PB2	ARG 389	3.03	B:ANP32A	ASP 191
A:PB2	ARG 369	2.54	B:ANP32A	ASP 193
A:PB2	ARG 368	3.24	B:ANP32A	ASP 193
A:PB2	ARG 142	3.27	B:ANP32A	GLU 199
A:PB2	ARG 142	2.64	B:ANP32A	GLU 201
A:PB2	GLY 729	3.84	B:ANP32A	GLY 217
A:PB2	LYS 440	3.45	B:ANP32A	GLU 226
A:PB2	SER 519	2.15	B:ANP32A	GLU 232
A:PB2	ARG702	2.76	B:ANP32A	TYR144
A:PB2	GLU627	3.34	B:ANP32A	PHE121
A:PB2	GLU 520	3.34	B:ANP32A	GLU 232
A:PB2	THR 521	3.67	B:ANP32A	GLU 232
A:PB2	ARG714	2.97	B:ANP32A	SER96
A:PB2	THR 329	2.72	B:ANP32A	GLN 235
A:PB2	TYR 55	2.60	B:ANP32A	ARG 12
A:PB2	PRO 56	3.77	B:ANP32A	ARG 12
A:PB2	SER 36	3.82	B:ANP32A	ASN 51
A:PB2	THR 215	3.32	B:ANP32A	GLN 184
A:PB2	ARG 213	3.90	B:ANP32A	VAL 186
A:PB2	SER 519	2.90	B:ANP32A	GLY 234
A:PB2	VAL 366	2.01	B:ANP32A	GLN 235
A:PB2	GLY 327	3.02	B:ANP32A	GLN 235
A:PB2	ASP 256	2.67	B:ANP32A	ARG 237
A:PB2	THR 303	3.35	B:ANP32A	ARG 237
A:PB2	PRO 302	2.86	B:ANP32A	ARG 237
A:PB2	GLU 487	2.71	B:ANP32A	LYS 238
A:PB2	ASP 253	3.01	B:ANP32A	ARG 239

Co-immunoprecipitation assays further demonstrated that the mutation M631L substantially enhanced ANP32A binding to PB2. The mutation E362G or D441N showed modest increases binding of ANP32A to PB2 although these differences did not reach statistical significance (Figure 3C).

### 3.4 Effect of PB2 mutations on polymerase activity

**Figure 4:**
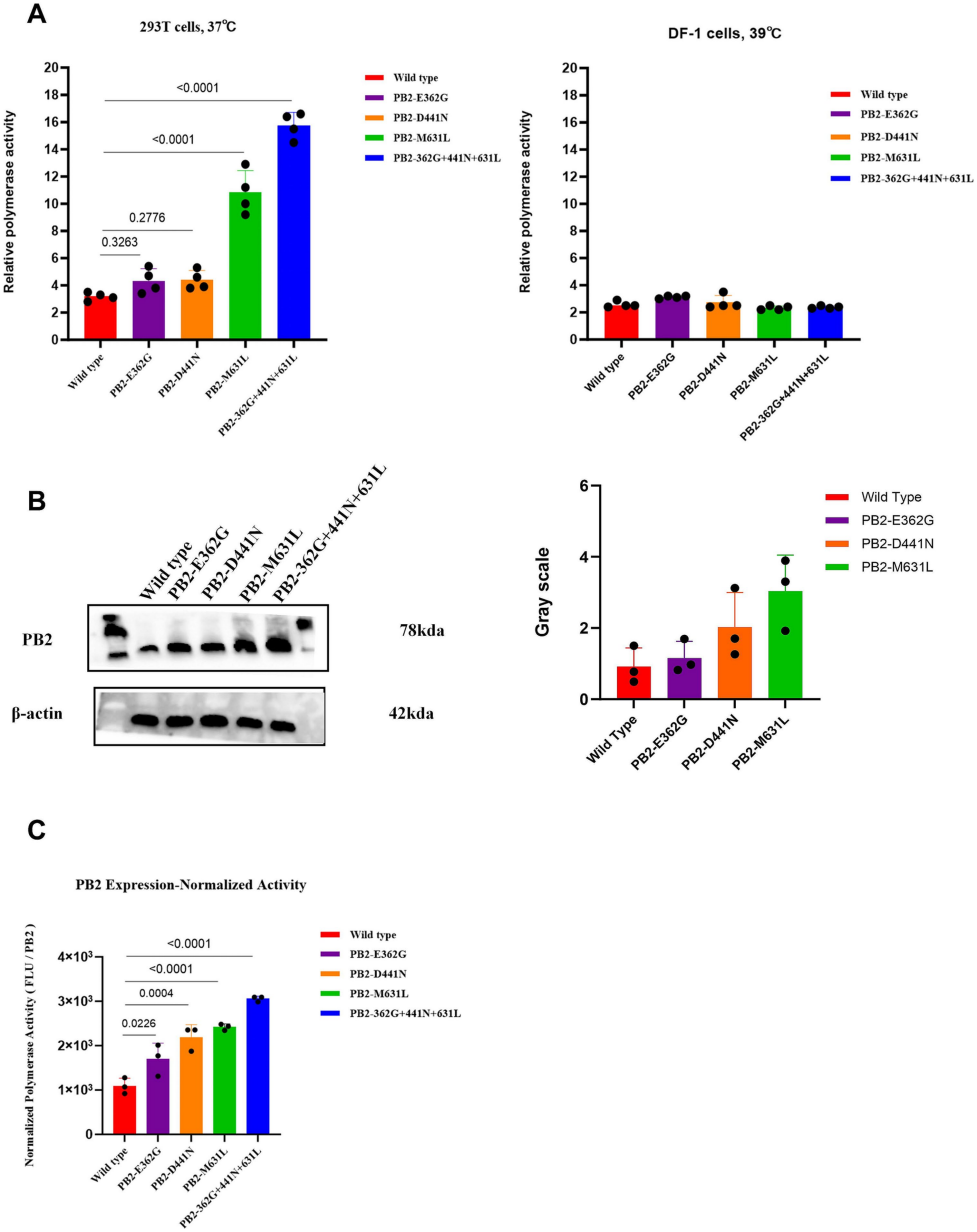
Effects of PB2 mutations on polymerase activity and protein expression. **(A)** Polymerase activity of either wild-type or mutant PB2 protein in HEK293T or DF-1 cells was measured and expressed as the ratio of firefly to Renilla luciferase activity, reported in relative luciferase units (RLU). **(B)** Western blot analysis was conducted to assess PB2 expression in 293 T cells transfected with plasmids encoding either wild-type or mutant PB2 proteins. Representative immunoblot images are presented on the left side. Densitometric quantification of PB2 protein levels, normalized to *β*-actin, is presented on the right. This analysis was conducted using ImageJ software and is based on data from three independent experiments. **(C)** The relative expression levels of the PB2 protein normalized to luciferase activity (Fluc). Statistical significance was determined by One-way (or Two-way) ANOVA followed by Dunnett’s post-hoc test for multiple comparisons. Error bars represent the mean ± standard deviation (SD) from three independent biological replicates. In accordance with the reviewer’s recommendation for optimal transparency, significance asterisks were replaced, and exact adjusted p-values (or specific intervals, such as *p* < 0.001) were directly annotated on the charts. Differences were considered statistically significant when the adjusted p-value was less than 0.05.

In human HEK293T cells, minigenome assays revealed that singular M631L but not E362G or D441N mutation significantly augmented polymerase activity. Strikingly, the triple-mutant constellation (E362G/D441N/M631L) exhibited functional levels far exceeding those of any individual variant. This supra-additive phenotype underscores a profound cooperative interplay that potentiates intracellular vRNP transcription and replication activity specifically within the human cellular environment. In sharp contrast, none of these mutations conferred a replicative advantage in avian DF-1 cells (Figure 4A). Western blot analysis of co-transfected 293 T cells showed that, except PB2-E362G mutation, PB2-D441N, PB2-M631L and the PB2-E362G/D441N/M631L mutant were expressed at significantly higher levels than the wild-type PB2 (Figure 4B). Further normalization of luciferase activity (Fluc) against the quantified relative PB2 protein expression levels(Figure 4C) demonstrated that the intrinsic enzymatic efficiencies of the PB2-M631L mutant and the triple mutant remained approximately 2.5- to 3-fold higher than that of the wild-type PB2.

Immunofluorescence staining further demonstrated that the intracellular distribution and nuclear localization patterns of the PB2 mutants were similar to those of the wild-type protein (Supplementary Figure S1), indicating that the observed enhancement in polymerase activity was not driven by altered nuclear trafficking.

### 3.5 Effects of PB2 protein mutations on viral replication kinetics

**Figure 5:**
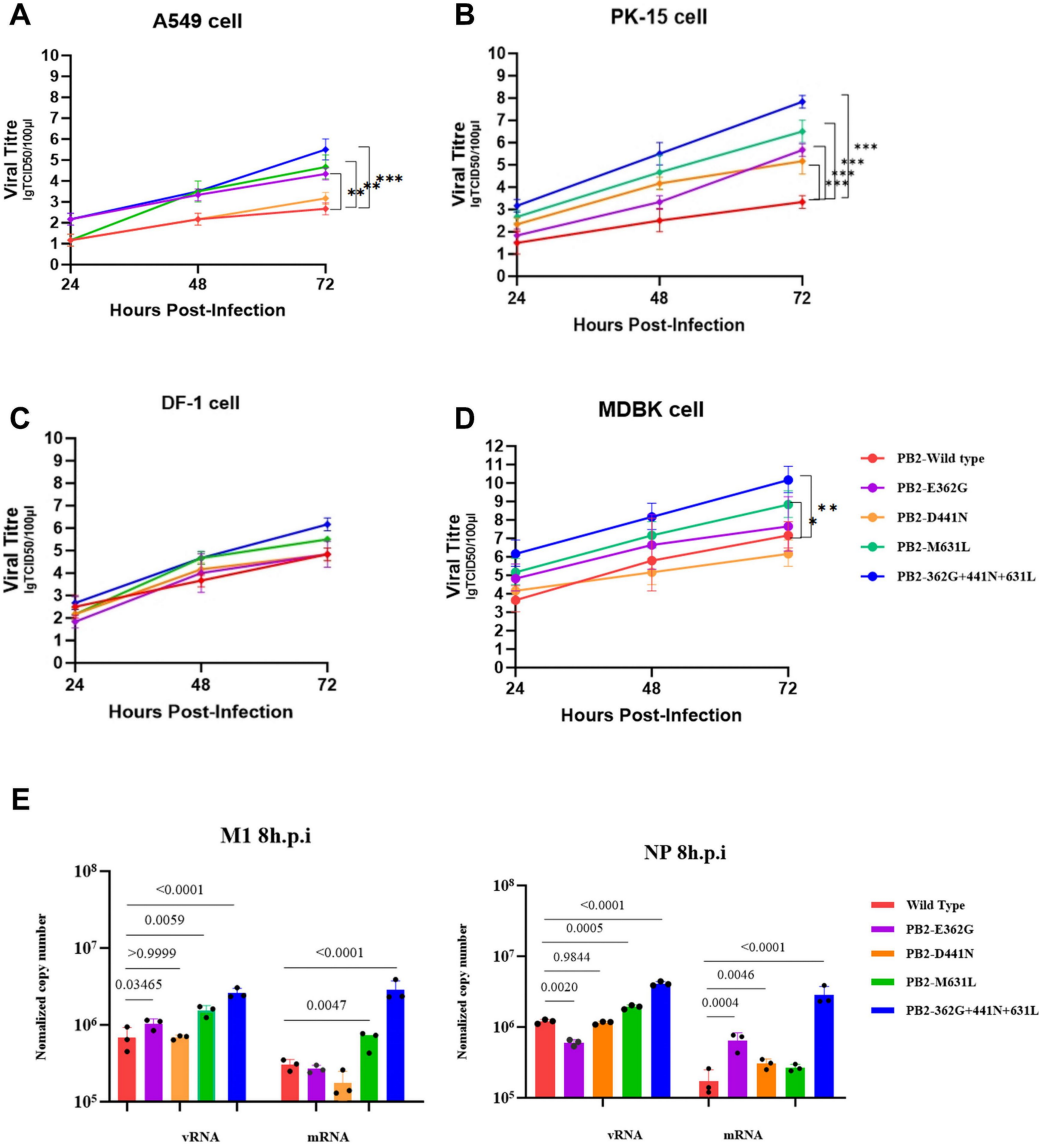
Replication kinetics of PB2-mutant H5N1 viruses in different host cell lines. H5N1 viruses harboring the specified PB2 substitutions infected A549 **(A)**, PK15 **(B)**, DF-1 **(C)**, and MDBK **(D)** cell lines at a multiplicity of infection (MOI) of 0.1. Supernatants were collected at 24, 48, and 72 h post-infection and viral titers were quantified using the TCID₅₀ assay. **(E)** Quantification of NP and M1 viral RNA (vRNA) and messenger RNA (mRNA) levels in A549 cells infected with PB2 mutant H5N1 viruses at a multiplicity of infection (MOI) of 0.1 was performed using RT-qPCR. Viral RNA levels are quantified as absolute copy numbers (copies per μg of total RNA), determined through standard curve analysis and normalized to β-actin expression. All statistical analyses and graph generation were performed using GraphPad Prism software (version 10). For comparisons involving multiple experimental groups, statistical significance was evaluated using One-way or Two-way analysis of variance (ANOVA). To strictly control the family-wise error rate during multiple testing, Dunnett’s *post-hoc* test was applied. Calculated multiple-comparison values were reported as adjusted *p*-values. In accordance with the reviewer’s recommendation for optimal transparency, significance asterisks were replaced, and exact adjusted *p*-values (or specific intervals, such as p < 0.001) were directly annotated on the charts. Differences were considered statistically significant when the adjusted *p*-value was less than 0.05.

To evaluate the replication kinetics of the mutant virus, this study infected A549, DF-1, PK-15, and MDBK cell lines at a multiplicity of infection (MOI) of 0.1. Virus-containing supernatants were collected at 24, 48 and 72 h post-infection, and viral titers were quantified using the 50% tissue culture infectious dose (TCID50) assay. The results showed that, compared to the wild-type virus, viruses bearing PB2-M631L or the combined mutations produced much higher titers in A549, PK-15 and MDBK cells, PB2-E362G mutant virus produce much higher titers in A549 and PK15 cells, and PB2-D441N mutant virus also produced much higher titer than the wild-type strain in A549 cells at 48 h or 72 h post infection whereas the titers has no significant difference in DF-1 cells between those mutant viruses (Figures 5A–D), suggesting that these substitutions confer a clear advantage in supporting viral replication in mammalian cells. Notably, PB2-E362G/D441N/M631L mutant virus, which presented the highest titers especially at 72 h post infection in those viruses, demonstrated the most robust replication advantage. In addition, the replication capacity of the PB2-D441N mutant in MDBK cells was slightly lower than that of the wild-type, a trend consistent with our observations in A549 cells. This suggests that the D441N mutation may require synergistic interactions with other mutations to confer optimal adaptive advantages.

Furthermore, fluorescent quantitative PCR were conducted to reveal the mRNA and vRNA levels of those mutant viruses in infected A549 at 8 h post-infection. The results showed that PB2-D441N mutant showed a significant reduction in mRNA levels of both NP and M1, whereas, vRNA levels of both NP and M1 remained unchanged. In contrast, the PB2-E362G mutant showed mild upregulation of vRNA of both M1 and NP, while mRNA levels remained unchanged. PB2-362G + 441 N + 631 L mutant virus presented significantly higher levels of vRNA or mRNA than each single mutant virus while the PB2-M631L or PB2-362G + 441 N + 631 L mutation significantly enhanced both vRNA and mRNA production (Figure 5E).

### 3.6 Pathogenicity differences in mice due to PB2 mutations

**Figure 6:**
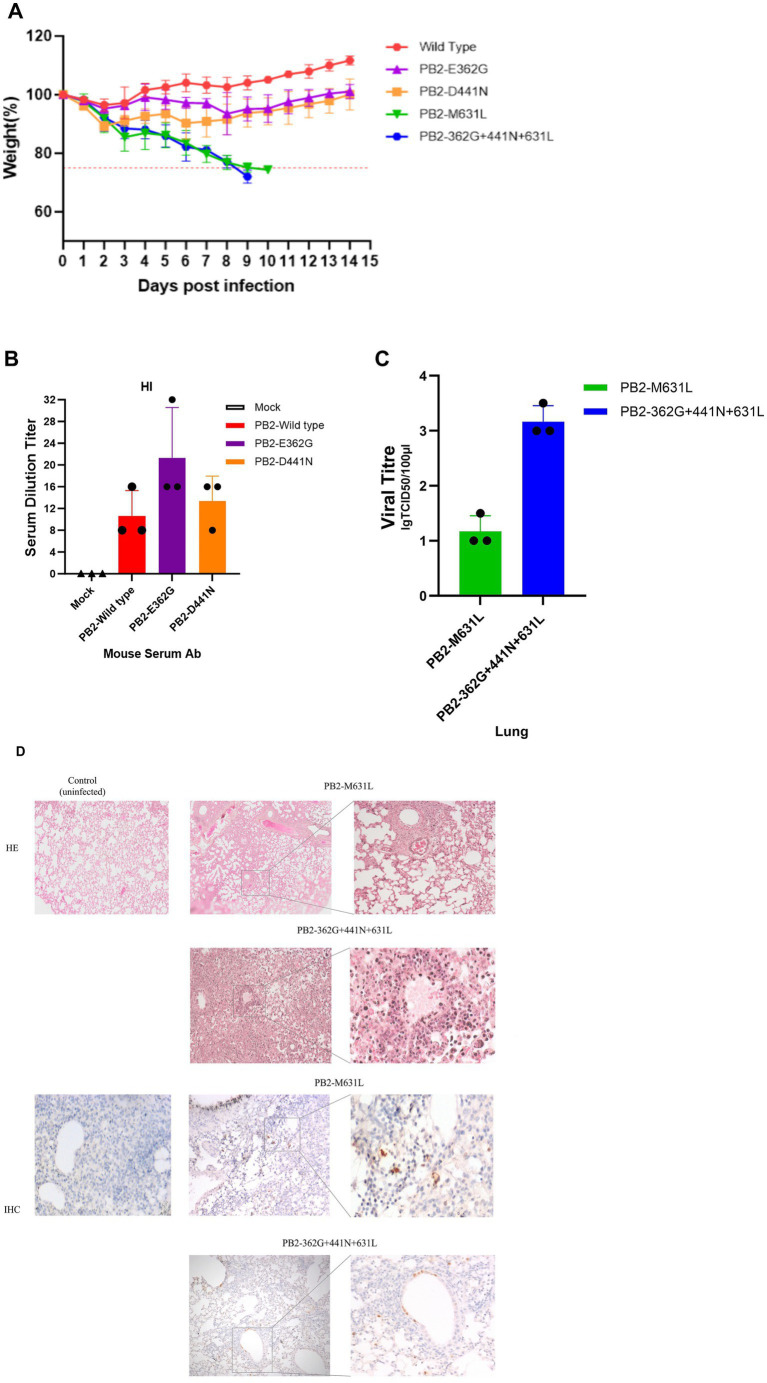
Pathogenicity of PB2-mutant H5N1 viruses in mice. **(A)** Body-weight changes of C57BL/6 J mice intranasally infected with wild-type or PB2-mutant virus. Body weight was monitored daily for 14 days (*n* = 3) and expressed as a percentage of the initial weight. **(B)** The bar graph illustrates the results of the hemagglutination inhibition (HI) assay conducted on mice infected with PB2 wild-type, PB2-E362G and PB2-D441N viral strains. **(C)** Viral TCID₅₀ titers in the lung tissue of mice infected with H5N1 viruses harboring the PB2-M631L mutation or the combined PB2-362G + 441 N + 631 L mutations (mean ± SD). **(D)** Hematoxylin and eosin (H&E) staining (top) and immunohistochemical analysis (bottom) were performed for histopathological evaluation and detection of nucleoprotein (NP) antigen in lung tissues of mice infected with H5N1 viruses harboring either the PB2-M631L mutation or the combined PB2-362G + 441 N + 631 L mutation. Representative images illustrate characteristic lesions and the distribution of viral antigens. Statistical significance was determined by One-way (or Two-way) ANOVA followed by Dunnett’s *post-hoc* test for multiple comparisons. Error bars represent the mean ± standard deviation (SD) from three independent biological replicates. In accordance with the reviewer’s recommendation for optimal transparency, significance asterisks were replaced, and exact adjusted *p*-values (or specific intervals, such as *p* < 0.001) were directly annotated on the charts. Differences were considered statistically significant when the adjusted *p*-value was less than 0.05.

To elucidate the effects of PB2 site mutations on viral infectivity and pathogenicity, the H5N1 mutants containing specific PB2 mutations were inoculated to infect mice. The results showed that the body weight presented significant decreased in mice infected with PB2-M631L or PB2-362G + 441 N + 631 L mutant virus, and all 3 mice infected with PB2-362G + 441 N + 631 L mutant virus and 2 mice with PB2-M631L infection died on day 9 post infection (another one died on day 10 post infection). In addition, PB2-E362G or PB2-D441N mutant virus infection resulted in weight loss throughout the infection period compared to PB2-wild virus infection, although body weight just presented a temporary loss in the mice infected with PB2-wild-type, PB2-E362G or PB2-D441N mutant virus (Figure 6A). Hemagglutination inhibition (HI) assays indicated that the specific antibodies can be induced by virus with PB2-E362G, PB2-D441N or PB2 wild type while no antibodies were detected in sera of PBS-inoculated mice, suggesting that all of those viruses can low pathogenically infected mice(Figure 6B). This aligns with the limited *in vitro* enhancement of polymerase activity. Viral titration showed that the viral TCID_50_ was much higher in in lung tissues harvested 9 days post-infection from combined mutant group than PB2-M631L single-mutant group (Figure 6C).

Hematoxylin and eosin (H&E) staining of lung tissues revealed that, in mice infected with the PB2-M631L mutant, most alveolar structures remained intact, although focal areas displayed toxic lung injury characterized by alveolar septal thickening, inflammatory cell infiltration, and hemorrhage. Lung sections from animals infected with the combined mutant virus exhibited characteristics of diffuse alveolar destruction, extensive inflammatory cell infiltration, and pulmonary consolidation (Figure 6D). Immunohistochemistry (IHC) staining revealed the presence of viral antigen–positive cells in both alveolar and bronchial epithelial cells across all infection groups in mice. In contrast to the triple-mutant group, the viral dispersion in the lungs of PB2-M631L–infected animals was more confined, with a limited number of bronchial epithelial cells displaying positive staining.

## 4 Discussion

While hemagglutinin (HA) adaptation is essential for viral entry, overcoming host restriction for efficient replication in mammals primarily relies on the viral polymerase PB2 subunit (Steel et al., 2009; Foeglein et al., 2011). In this study, we identified three PB2 substitutions—E362G, D441N, and M631L—as key drivers of mammalian adaptation in H5N1 viruses and elucidated their molecular mechanisms. Evolutionary analysis revealed that the PB2-E362G, PB2-D441N, and PB2-M631L mutations have attained high prevalence in recent mammalian-derived isolates and exert measurable functional impacts. Site-specific entropy analysis revealed elevated variability at these positions (E362G: 0.461, D441N: 0.451, M631L: 0.450). A higher entropy score indicates a greater diversity of amino acids and reduced sequence conservation at these positions, reflecting that these residues possess a high degree of evolutionary plasticity during viral circulation in mammalian hosts. Temporally, these substitutions rapidly accumulated and reached high prevalence (>95%) in dairy cattle isolates by 2024, whereas their prevalence in avian reservoirs remained negligible. The concomitant emergence and distinct host-stratified distribution of these substitutions suggest they are not stochastic variations but represent critical adaptive signatures driving the establishment of H5N1 in mammalian hosts.

Mechanistically, the adaptive advantage of these mutations appears to be linked to enhanced interactions with mammalian host factors. Our structural analysis suggests that PB2-E362G may modulate cap-binding activity by altering the local aromatic stacking, thereby influencing viral transcription initiation (Nataraj et al., 2025; Carrique et al., 2020). More critically, the M631L mutation within the 627–631 loop likely enhances the stability of the polymerase-ANP32A complex by optimizing the “positive charge ridge” at the binding interface (Camacho-Zarco et al., 2020). Furthermore, species-specific differences in the ANP32 family are critical for interpreting our findings, human ANP32A and ANP32B are essential but redundant host factors for influenza virus genome replication (Staller et al., 2019). Previous studies have demonstrated that murine ANP32A cannot support avian influenza polymerase activity; thus, viral replication in mice relies primarily on ANP32B (Zhang et al., 2019). ANP32A and ANP32B exhibit high structural similarity and charge properties in the C-terminal acidic tail (LCAR), a key region for binding to PB2 (Carrique et al., 2020; Camacho-Zarco et al., 2020). Residing on the exposed surface of the PB2 627 domain, the M631L mutation increases local hydrophobicity. This change was predicted to optimize the binding interface with the acidic tails of host ANP32 proteins or, alternatively, boost polymerase activity directly, ultimately conferring a high-virulence phenotype.

Integration of *in vitro* polymerase assays, cellular replication kinetics, and murine infection models for the functional and pathogenic characterization of mutant H5N1 viruses demonstrates that PB2-M631L serves as the primary determinant driving enhanced viral replication and pathogenicity in mammalian systems. This substitution significantly augments both polymerase activity and viral genomic replication efficiency in vitro. Importantly, these findings suggest that the mutation confers a significant qualitative enhancement to the intrinsic enzymatic efficiency and host-factor compatibility of the polymerase complex in mammalian cells, which cannot be solely attributed to a quantitative increase in PB2 protein expression levels. This substitution significantly upregulated polymerase activity and viral RNA synthesis in vitro, suggesting that PB2-M631L facilitates host adaptation by optimizing the functional compatibility of the viral polymerase complex with mammalian host factors. In addition, although viruses harboring single PB2-E362G or PB2-D441N substitution exhibited an attenuated phenotype characterized by transient weight loss, serological analysis via HI assays confirmed robust seroconversion in these animals. This demonstrates that while these single-substitution variants remain infectious and capable of eliciting an immune response, they lack the virulence required to cause severe morbidity compared to the M631L variant. Consistently, the triple-mutant combination exhibits significant cumulative effect, demonstrating enhanced polymerase activity and higher replication efficiency in mammalian cells. And the combined effect also was demonstrated in mice infection results shown by higher viral load, more viral distribution, and more severe diffuse alveolar damage and pulmonary consolidation in lungs of mouse infected with combined mutant virus than the PB2-M631L single mutant virus. This indicated that the combined mutations confer enhanced replication. The potential mechanism of the significant cumulative effect may be attributed to PB2-M631L functioning as the primary differentiating factor. In this context, while PB2-M631L functions as the primary phenotypic driver, PB2-D441N appears to act as an accessory or modulatory mutation. Singularly, PB2-E362G and PB2-D441N confers limited functional advantages across different cell lines; however, within the genetic background of M631L, it likely facilitates subtle structural adjustments or enhances RNP stability, thereby playing a supportive role in promoting the early replication process. The conclusions of this study corroborate those of the 2025 research conducted by Bayoumi et al. The study in question compared human and bovine influenza virus strains, and its findings indicated that PB2-M63I and E627K exhibited a cooperative mutation that augmented the polymerase activity in mammalian cells, particularly those of bovine origin (Caserta et al., 2024; Camacho-Zarco et al., 2020; Gu et al., 2024; Zhang et al., 2017).

In summary, this study elucidates the molecular basis and functional characteristics of the PB2 protein in the mammalian adaptation of the highly pathogenic H5N1 B3.13 lineage. Evolutionary analysis reveals that the PB2-E362G, PB2-D441N, and PB2-M631L mutations emerged concurrently, attained high prevalence, and persisted in mammalian-derived isolates, thereby exerting distinct functional roles. Among these, PB2-M631L stands out as the primary determinant associated with elevated polymerase activity, replication efficiency, and mouse pathogenicity, underscoring its role as a key molecular marker of mammalian host restriction. Furthermore, the co-occurrence of PB2-E362G and PB2-D441N with PB2-M631L exerts a combined effect that further amplifies intracellular polymerase activity, thereby optimizing the mechanisms of intracellular transcription and replication during viral adaptation to mammalian hosts.

Several limitations of the present study should be acknowledged. First, although the mouse model serves as a well-established surrogate for assessing mammalian pathogenicity, caution should be exercised when extrapolating these findings across species, as host factors—particularly species-specific differences in ANP32A isoforms—may differentially modulate polymerase function in humans or cattle. Nonetheless, we acknowledge several key limitations in our study. Second, from a methodological perspective, we explicitly note that while our densitometric normalization confirms a qualitative enhancement in intrinsic polymerase efficiency, the overall robust increase in polymerase activity observed in our initial assays is likely influenced, at least in part, by the higher levels of steady-state PB2 protein expression in these mutants. Finally, our study did not include direct inter-mammalian transmission experiments, such as aerosol or contact transmission assays in relevant animal models. Therefore, the enhanced intracellular replication and murine virulence demonstrated here do not automatically imply increased transmissibility, which is a highly complex phenotype requiring further verification in transmission-competent models.

## Data Availability

The original contributions presented in the study are included in the article/Supplementary material, further inquiries can be directed to the corresponding authors.

## Footnotes

1
https://nextstrain.org/

2
https://alphafoldserver.com/

3
https://swissmodel.expasy.org/

4
http://hdock.phys.hust.edu.cn/

## References

[ref1] Burrough E. R. Magstadt D. R. Petersen B. Timmermans S. J. Gauger P. C. Zhang J. . (2024). Highly pathogenic avian influenza a(H5N1) clade 2.3.4.4b virus infection in domestic dairy cattle and cats, United States, 2024. Emerg. Infect. Dis. 30, 1335–1343. doi: 10.3201/eid3007.240508, 38683888 PMC11210653

[ref2] Bussey K. A. Bousse T. L. Desmet E. A. Kim B. Takimoto T. (2010). PB2 residue 271 plays a key role in enhanced polymerase activity of influenza a viruses in mammalian host cells. J. Virol. 84, 4395–4406. doi: 10.1128/JVI.02642-09, 20181719 PMC2863787

[ref3] Caliendo V. Lewis N. S. Pohlmann A. Baillie S. R. Banyard A. C. Beer M. . (2022). Transatlantic spread of highly pathogenic avian influenza H5N1 by wild birds from Europe to North America in 2021. Sci. Rep. 12:11729. doi: 10.1038/s41598-022-13447-z, 35821511 PMC9276711

[ref4] Camacho-Zarco A. R. Kalayil S. Maurin D. Salvi N. Delaforge E. Milles S. . (2020). Molecular basis of host-adaptation interactions between influenza virus polymerase PB2 subunit and ANP32A. Nat. Commun. 11:3656. doi: 10.1038/s41467-020-17407-x, 32694517 PMC7374565

[ref5] Carrique L. Fan H. Walker A. P. Keown J. R. Sharps J. Staller E. . (2020). Host ANP32A mediates the assembly of the influenza virus replicase. Nature 587, 638–643. doi: 10.1038/s41586-020-2927-z, 33208942 PMC7116770

[ref6] Caserta L. C. Frye E. A. Butt S. L. Laverack M. Nooruzzaman M. Covaleda L. M. . (2024). Spillover of highly pathogenic avian influenza H5N1 virus to dairy cattle. Nature 634, 669–676. doi: 10.1038/s41586-024-07849-4, 39053575 PMC11485258

[ref7] De Graaf M. Fouchier R. A. (2014). Role of receptor binding specificity in influenza a virus transmission and pathogenesis. EMBO J. 33, 823–841. doi: 10.1002/embj.201387442, 24668228 PMC4194109

[ref8] Eisfeld A. J. Biswas A. Guan L. Gu C. Maemura T. Trifkovic S. . (2024). Pathogenicity and transmissibility of bovine H5N1 influenza virus. Nature 633, 426–432. doi: 10.1038/s41586-024-07766-6, 38977017 PMC11390473

[ref9] Fabrizio T. P. Kandeil A. Harrington W. N. Jones J. C. Jeevan T. Andreev K. . (2025). Genotype B3.13 influenza a(H5N1) viruses isolated from dairy cattle demonstrate high virulence in laboratory models, but retain avian virus-like properties. Nat. Commun. 16:6771. doi: 10.1038/s41467-025-61757-3, 40695800 PMC12284218

[ref10] Foeglein A. Loucaides E. M. Mura M. Wise H. M. Barclay W. S. Digard P. (2011). Influence of PB2 host-range determinants on the intranuclear mobility of the influenza a virus polymerase. J. Gen. Virol. 92, 1650–1661. doi: 10.1099/vir.0.031492-0, 21471313 PMC3167894

[ref11] Fouchier R. A. Schneeberger P. M. Rozendaal F. W. Broekman J. M. Kemink S. A. Munster V. . (2004). Avian influenza a virus (H7N7) associated with human conjunctivitis and a fatal case of acute respiratory distress syndrome. Proc. Natl. Acad. Sci. USA 101, 1356–1361. doi: 10.1073/pnas.0308352100, 14745020 PMC337057

[ref12] Gu C. Maemura T. Guan L. Eisfeld A. J. Biswas A. Kiso M. . (2024). A human isolate of bovine H5N1 is transmissible and lethal in animal models. Nature 636, 711–718. doi: 10.1038/s41586-024-08254-7, 39467571 PMC12629513

[ref13] Hatta M. Gao P. Halfmann P. Kawaoka Y. (2001). Molecular basis for high virulence of Hong Kong H5N1 influenza a viruses. Science 293, 1840–1842. doi: 10.1126/science.1062882, 11546875

[ref14] Hu J. Mo Y. Wang X. Gu M. Hu Z. Zhong L. . (2015). PA-X decreases the pathogenicity of highly pathogenic H5N1 influenza a virus in avian species by inhibiting virus replication and host response. J. Virol. 89, 4126–4142. doi: 10.1128/JVI.02132-14, 25631083 PMC4442343

[ref15] Imai M. Herfst S. Sorrell E. M. Schrauwen E. J. Linster M. De Graaf M. . (2013). Transmission of influenza a/H5N1 viruses in mammals. Virus Res. 178, 15–20. doi: 10.1016/j.virusres.2013.07.017, 23954580 PMC3838911

[ref16] Jahid M. J. Nolting J. M. (2025). Dynamics of a panzootic: genomic insights, host range, and epidemiology of the highly pathogenic avian influenza a(H5N1) clade 2.3.4.4b in the United States. Viruses 17:312. doi: 10.3390/v17030312, 40143242 PMC11946527

[ref17] Le Q. M. Ito M. Muramoto Y. Hoang P. V. Vuong C. D. Sakai-Tagawa Y. . (2003). Pathogenicity of highly pathogenic avian H5N1 influenza a viruses isolated from humans between 2003 and 2008 in northern Vietnam. J. Gen. Virol. 91, 2485–2490. doi: 10.1099/vir.0.021659-0, 20592108 PMC3052597

[ref18] Lee D. H. Torchetti M. K. Winker K. Ip H. S. Song C. S. Swayne D. E. (2015). Intercontinental spread of Asian-origin H5N8 to North America through Beringia by migratory birds. J. Virol. 89, 6521–6524. doi: 10.1128/JVI.00728-15, 25855748 PMC4474297

[ref19] Leguia M. Garcia-Glaessner A. Munoz-Saavedra B. Juarez D. Barrera P. Calvo-Mac C. . (2023). Highly pathogenic avian influenza a (H5N1) in marine mammals and seabirds in Peru. Nat. Commun. 14:5489. doi: 10.1038/s41467-023-41182-0, 37679333 PMC10484921

[ref20] Lewis N. S. Banyard A. C. Whittard E. Karibayev T. Al Kafagi T. Chvala I. . (2021). Emergence and spread of novel H5N8, H5N5 and H5N1 clade 2.3.4.4 highly pathogenic avian influenza in 2020. Emerg. Microbes Infect. 10, 148–151. doi: 10.1080/22221751.2021.1872355, 33400615 PMC7832535

[ref21] Long J. S. Giotis E. S. Moncorge O. Frise R. Mistry B. James J. . (2016). Species difference in ANP32A underlies influenza a virus polymerase host restriction. Nature 529, 101–104. doi: 10.1038/nature16474, 26738596 PMC4710677

[ref22] Mistry B. Long J. S. Schreyer J. Staller E. Sanchez-David R. Y. Barclay W. S. (2020). Elucidating the interactions between influenza virus polymerase and host factor ANP32A. J. Virol. 94:e01353–19. doi: 10.1128/JVI.01353-19, 31694956 PMC7000967

[ref23] Moncorge O. Mura M. Barclay W. S. (2010). Evidence for avian and human host cell factors that affect the activity of influenza virus polymerase. J. Virol. 84, 9978–9986. doi: 10.1128/JVI.01134-10, 20631125 PMC2937815

[ref24] Moreno A. Bonfante F. Bortolami A. Cassaniti I. Caruana A. Cottini V. . (2023). Asymptomatic infection with clade 2.3.4.4b highly pathogenic avian influenza a(H5N1) in carnivore pets, Italy, April 2023. Euro Surveill. 28:2300441. doi: 10.2807/1560-7917.ES.2023.28.35.2300441, 37650905 PMC10472752

[ref25] Mostafa A. Naguib M. M. Nogales A. Barre R. S. Stewart J. P. Garcia-Sastre A. . (2024). Avian influenza a (H5N1) virus in dairy cattle: origin, evolution, and cross-species transmission. MBio 15:e0254224. doi: 10.1128/mbio.02542-24, 39535188 PMC11633217

[ref26] Nataraj R. Ashok A. K. Dey A. A. Kesavardhana S. (2025). Decoding non-human mammalian adaptive signatures of 2.3.4.4b H5N1 to assess its human adaptive potential. Microbiol. Spectrum 13:e0094825. doi: 10.1128/spectrum.00948-25, 40788041 PMC12403652

[ref27] Nelli R. K. Harm T. A. Siepker C. Groeltz-Thrush J. M. Jones B. Twu N. C. . (2024). Sialic acid receptor specificity in mammary gland of dairy cattle infected with highly pathogenic avian influenza a(H5N1) virus. Emerg. Infect. Dis. 30, 1361–1373. doi: 10.3201/eid3007.240689, 38861554 PMC11210646

[ref28] Resa-Infante P. Gabriel G. (2013). The nuclear import machinery is a determinant of influenza virus host adaptation. BioEssays 35, 23–27. doi: 10.1002/bies.201200138, 23239226

[ref29] Restori K. H. Septer K. M. Field C. J. Patel D. R. Vaninsberghe D. Raghunathan V. . (2024). Risk assessment of a highly pathogenic H5N1 influenza virus from mink. Nat. Commun. 15:4112. doi: 10.1038/s41467-024-48475-y, 38750016 PMC11096306

[ref30] Salomon R. Franks J. Govorkova E. A. Ilyushina N. A. Yen H. L. Hulse-Post D. J. . (2006). The polymerase complex genes contribute to the high virulence of the human H5N1 influenza virus isolate a/Vietnam/1203/04. J. Exp. Med. 203, 689–697. doi: 10.1084/jem.20051938, 16533883 PMC2118237

[ref31] Shinya K. Hamm S. Hatta M. Ito H. Ito T. Kawaoka Y. (2004). PB2 amino acid at position 627 affects replicative efficiency, but not cell tropism, of Hong Kong H5N1 influenza a viruses in mice. Virology 320, 258–266. doi: 10.1016/j.virol.2003.11.030, 15016548

[ref32] Staller E. Sheppard C. M. Neasham P. J. Mistry B. Peacock T. P. Goldhill D. H. . (2019). ANP32 proteins are essential for influenza virus replication in human cells. J. Virol. 93:e00217–19. doi: 10.1128/JVI.00217-19, 31217244 PMC6694824

[ref33] Steel J. Lowen A. C. Mubareka S. Palese P. (2009). Transmission of influenza virus in a mammalian host is increased by PB2 amino acids 627K or 627E/701N. PLoS Pathog. 5:e1000252. doi: 10.1371/journal.ppat.1000252, 19119420 PMC2603332

[ref34] Uhart M. M. Vanstreels R. E. T. Nelson M. I. Olivera V. Campagna J. Zavattieri V. . (2024). Epidemiological data of an influenza a/H5N1 outbreak in elephant seals in Argentina indicates mammal-to-mammal transmission. Nat. Commun. 15:9516. doi: 10.1038/s41467-024-53766-5, 39528494 PMC11555070

[ref35] Webby R. J. Webster R. G. (2003). Are we ready for pandemic influenza? Science 302, 1519–1522. doi: 10.1126/science.1090350, 14645836

[ref36] Welkers M. R. A. Pawestri H. A. Fonville J. M. Sampurno O. D. Pater M. Holwerda M. . (2019). Genetic diversity and host adaptation of avian H5N1 influenza viruses during human infection. Emerg. Microbes Infect. 8, 262–271. doi: 10.1080/22221751.2019.1575700, 30866780 PMC6455201

[ref37] Zhang X. Xu G. Wang C. Jiang M. Gao W. Wang M. . (2017). Enhanced pathogenicity and neurotropism of mouse-adapted H10N7 influenza virus are mediated by novel PB2 and NA mutations. J. Gen. Virol. 98, 1185–1195. doi: 10.1099/jgv.0.000770, 28597818

[ref38] Zhang H. Zhou L. Amichai S. Zandi K. Cox B. Schinazi R. . (2019). Novel influenza polymerase PB2 inhibitors for the treatment of influenza A infection. Bioorganic & Medicinal Chemistry Letters 29:126639. doi: 10.1016/j.bmcl.2019.126639, 31493987 PMC7732027

[ref39] Zhou B. Li Y. Halpin R. Hine E. Spiro D. J. Wentworth D. E. (2011). PB2 residue 158 is a pathogenic determinant of pandemic H1N1 and H5 influenza a viruses in mice. J. Virol. 85, 357–365. doi: 10.1128/JVI.01694-10, 20962098 PMC3014153

