# Supplementary material for: Identification and characterization of PB2 mutations associated with mammalian adaptation of highly pathogenic H5N1 avian influenza viruses
Source: Front Microbiol. 2026 Jul 29;17:1867604. doi: 10.3389/fmicb.2026.1867604 (PMC13461586; doi:10.3389/fmicb.2026.1867604)
Supplement: Supplementary file 1 [file Table_1.docx]

Supplementary Material

# 1 Supplementary Figures and Tables

**Supplementary S1**

Supplementary S2. Original Western blot images

Original Table

Supplementary Table 1 Dual-luciferase reporter assay of viral polymerase activities in human 293T and DF-1 cells.

| **(Firefly)+(Renilla)** | | | | | |
| --- | --- | --- | --- | --- | --- |
| 293T | | | | | |
| GROUP | WT | PB2-362G | PB2-D441N | PB2-M631L | PB2-362G+441N+631L |
| Replicate 1 (Firefly) | 12104 | 12437 | 10321 | 18200 | 32760 |
| 1 (Renilla) | 3633 | 2298 | 2271 | 1831 | 2120 |
| Replicate 2 (Firefly) | 14688 | 14720 | 14711 | 15377 | 29490 |
| 2 (Renilla) | 4242 | 3136 | 2786 | 1377 | 1650 |
| Replicate 3 (Firefly) | 14390 | 13810 | 16380 | 18260 | 33540 |
| 3 (Renilla) | 5109 | 4020 | 4168 | 1981 | 2050 |
| Replicate 4 (Firefly) | 13710 | 12980 | 12710 | 18870 | 29660 |
| 4 (Renilla) | 4475 | 3407 | 3393 | 1459 | 2042 |
| DF-1 | | | | | |
| Replicate 1 (Firefly) | 7339 | 7468 | 6985 | 7722 | 7298 |
| 1 (Renilla) | 2860 | 2440 | 2020 | 3490 | 3090 |
| Replicate 2 (Firefly) | 6391 | 6558 | 6541 | 6709 | 6592 |
| 2 (Renilla) | 2650 | 2060 | 2790 | 2740 | 2640 |
| Replicate 3 (Firefly) | 6282 | 6147 | 6500 | 6428 | 6749 |
| 3 (Renilla) | 2150 | 2150 | 2620 | 2680 | 2920 |
| Replicate 4 (Firefly) | 6169 | 6169 | 6519 | 6756 | 6803 |
| 4 (Renilla) | 2460 | 2460 | 2650 | 3120 | 2980 |

Supplementary Table 2 Viral replication kinetics of H5N1 recombinant viruses in A549,PK-15,DF-1, and MDBK cells.

| **A549** | | | | | | | | | | | | | | | |
| --- | --- | --- | --- | --- | --- | --- | --- | --- | --- | --- | --- | --- | --- | --- | --- |
| dpi | PB2-Wild Type | | | PB2-E362G | | | PB2-D441N | | | PB2-M631L | | | PB2-362G+441N+631L | | |
| 24 | 1 | 1.5 | 1 | 2 | 2 | 2.5 | 1 | 1.5 | 1 | 1.5 | 1 | 1 | 2 | 2.5 | 2 |
| 48 | 2 | 2 | 2.5 | 3 | 3.5 | 3.5 | 2 | 2 | 2.5 | 3 | 3.5 | 4 | 3.5 | 3.5 | 3.5 |
| 72 | 2.5 | 2.5 | 3 | 4.5 | 4 | 4.5 | 3.5 | 3 | 3 | 5 | 4 | 5 | 5.5 | 6 | 5 |
| **PK-15** | | | | | | | | | | | | | | | |
| dpi | PB2-Wild Type | | | PB2-E362G | | | PB2-D441N | | | PB2-M631L | | | PB2-362G+441N+631L | | |
| 24 | 1 | 1.5 | 2 | 2 | 1.5 | 2 | 2 | 2.5 | 2.5 | 3 | 2.5 | 2.5 | 3 | 3.5 | 3 |
| 48 | 2 | 3 | 2.5 | 3 | 3.5 | 3.5 | 4.5 | 4 | 4 | 4 | 4.5 | 5.5 | 5 | 5.5 | 6 |
| 72 | 3.5 | 3.5 | 3 | 5.5 | 5.5 | 6 | 5.5 | 4.5 | 5.5 | 6.5 | 6 | 7 | 7.5 | 8 | 8 |

| **DF-1** | | | | | | | | | | | | | | | | | | | | | | | |
| --- | --- | --- | --- | --- | --- | --- | --- | --- | --- | --- | --- | --- | --- | --- | --- | --- | --- | --- | --- | --- | --- | --- | --- |
| dpi | PB2-Wild Type | | | | PB2-E362G | | | | | PB2-D441N | | | | PB2-M631L | | | | | PB2-362G+441N+631L | | | | |
| 24 | 2 | | 2.5 | 3 | 2 | | 1.5 | | 2 | 2 | 2.5 | 2 | | 2 | | 2 | 2.5 | | 2.5 | | 3 | | 2.5 |
| 48 | 3.5 | | 3.5 | 4 | 3 | | 4.5 | | 4.5 | 4 | 4.5 | 4 | | 4.5 | | 4.5 | 5 | | 4.5 | | 4.5 | | 5 |
| 72 | 5 | | 4.5 | 5 | 4.5 | | 5.5 | | 4.5 | 4.5 | 5 | 5 | | 5.5 | | 5.5 | 5.5 | | 6 | | 6.5 | | 6 |
| **MDBK** | | | | | | | | | | | | | | | | | | | | | | | |
| dpi | | PB2-Wild Type | | | | PB2-E362G | | | | PB2-D441N | | | | | PB2-M631L | | | PB2-362G+441N+631L | | | | | |
| 24 | | 4 | 3.5 | 3.5 | | 5 | | 5 | 4.5 | 4.5 | 4 | | 4 | | 5 | 5.5 | 5 | 6 | | 6 | | 6.5 | |
| 48 | | 5 | 6.5 | 6 | | 7 | | 6 | 7 | 5 | 5.5 | | 5 | | 7 | 7.5 | 7 | 8 | | 8 | | 8.5 | |
| 72 | | 7 | 7.5 | 7 | | 8 | | 7 | 8 | 6 | 6.5 | | 6 | | 9 | 8.5 | 9 | 10 | | 10 | | 10.5 | |

Supplementary Table 3 Viral titers of recombinant H5N1 viruses in mammalian and avian cell lines at multiple time points

| **M1 VRNA** | | | | |
| --- | --- | --- | --- | --- |
| Wild type | PB2-362G | PB2-D441N | PB2-M631L | PB2-362G+441N+631L |
| 450000 | 1150000 | 709000 | 1610000 | 3050000 |
| 939000 | 1110000 | 638000 | 1250000 | 2410000 |
| 646000 | 845000 | 721000 | 1730000 | 2340000 |
| M1 mRNA | | | | |
| Wild type | PB2-362G | PB2-D441N | PB2-M631L | PB2-362G+441N+631L |
| 140000 | 430000 | 350000 | 260000 | 3890000 |
| 130000 | 730000 | 250000 | 300000 | 2310000 |
| 260000 | 770000 | 310000 | 240000 | 2360000 |
|  |  |  |  |  |
| NP VRNA | | | | |
| Wild type | PB2-362G | PB2-D441N | PB2-M631L | PB2-362G+441N+631L |
| 1110000 | 536000 | 1180000 | 1940000 | 4030000 |
| 1210000 | 620000 | 1090000 | 2040000 | 4430000 |
| 1280000 | 657000 | 1190000 | 1780000 | 3890000 |
| NP MRNA | | | | |
| Wild type | PB2-362G | PB2-D441N | PB2-M631L | PB2-362G+441N+631L |
| 140000 | 430000 | 350000 | 260000 | 3890000 |
| 120000 | 730000 | 250000 | 300000 | 2310000 |
| 260000 | 770000 | 310000 | 240000 | 2360000 |

Supplementary Table 4 Percentage of initial body weight in BALB/c mice infected with recombinant H5N1 viruses

| **Body-weight** | | | | | | | | | | | | | | | |
| --- | --- | --- | --- | --- | --- | --- | --- | --- | --- | --- | --- | --- | --- | --- | --- |
| 0 | 100 | 100 | 100 | 100 | 100 | 100 | 100 | 100 | 100 | 100 | 100 | 100 | 100 | 100 | 100 |
| 1 | 98.7804878 | 98.06451613 | 98.22485207 | 98.17073171 | 98.21428571 | 97.2972973 | 96.59090909 | 95.80838323 | 96 | 94.88636364 | 100 | 98.125 | 98.1595092 | 98.30508475 | 98.28571429 |
| 2 | 98.17073171 | 94.19354839 | 97.04142012 | 96.95121951 | 95.23809524 | 93.51351351 | 88.06818182 | 91.01796407 | 88.57142857 | 90.34090909 | 97.70114943 | 88.125 | 92.02453988 | 87.57062147 | 97.14285714 |
| 3 | 99.3902439 | 90.96774194 | 101.183432 | 96.95121951 | 94.64285714 | 97.2972973 | 90.34090909 | 89.82035928 | 93.14285714 | 88.63636364 | 87.93103448 | 80 | 84.66257669 | 89.26553672 | 91.42857143 |
| 4 | 104.2682927 | 100 | 100.591716 | 103.6585366 | 94.64285714 | 98.91891892 | 89.77272727 | 94.01197605 | 94.28571429 | 91.47727273 | 88.50574713 | 80.625 | 84.66257669 | 90.96045198 | 88.57142857 |
| 5 | 103.6585366 | 100 | 104.1420118 | 101.2195122 | 94.64285714 | 98.91891892 | 93.18181818 | 96.40718563 | 90.85714286 | 88.06818182 | 89.08045977 | 81.25 | 81.59509202 | 88.70056497 | 87.42857143 |
| 6 | 107.3170732 | 101.2903226 | 103.5502959 | 97.56097561 | 95.23809524 | 98.91891892 | 88.63636364 | 96.40718563 | 85.71428571 | 85.79545455 | 86.20689655 | 78.75 | 77.3006135 | 87.00564972 | 82.28571429 |
| 7 | 106.097561 | 100.6451613 | 102.9585799 | 97.56097561 | 95.23809524 | 98.37837838 | 87.5 | 97.00598802 | 88 | 82.95454545 | 78.73563218 | 77.5 | 79.75460123 | 81.3559322 | 82.28571429 |
| 8 | 104.2682927 | 98.70967742 | 104.7337278 | 97.56097561 | 85.11904762 | 97.83783784 | 85.79545455 | 95.80838323 | 93.14285714 | 79.54545455 | 74.71264368 | 76.25 | 77.3006135 | 79.0960452 | 74.85714286 |
| 9 | 106.7073171 | 101.9354839 | 103.5502959 | 98.17073171 | 90.47619048 | 96.75675676 | 88.63636364 | 98.20359281 | 94.28571429 | 76.70454545 | 73.56321839 | 75 | 70.55214724 | 74.57627119 | 70.85714286 |
| 10 | 105.4878049 | 104.516129 | 105.3254438 | 96.95121951 | 89.88095238 | 98.91891892 | 89.20454545 | 98.20359281 | 95.42857143 | 74.345289 |  |  |  |  |  |
| 11 | 107.9268293 | 105.8064516 | 107.1005917 | 97.56097561 | 93.45238095 | 101.6216216 | 89.20454545 | 100 | 97.14285714 |  |  |  |  |  |  |
| 12 | 110.3658537 | 105.8064516 | 107.6923077 | 96.95121951 | 97.61904762 | 102.1621622 | 91.47727273 | 100 | 98.85714286 |  |  |  |  |  |  |
| 13 | 110.9756098 | 107.7419355 | 111.2426036 | 100.6097561 | 98.80952381 | 101.6216216 | 93.18181818 | 101.1976048 | 99.42857143 |  |  |  |  |  |  |
| 14 | 113.4146341 | 110.9677419 | 110.6508876 | 100 | 99.4047619 | 103.7837838 | 94.31818182 | 104.7904192 | 101.1428571 |  |  |  |  |  |  |

Supplementary Table 5 HI antibody titers in mice infected with recombinant H5N1 viruses

| **(HI) assay results** | | | |
| --- | --- | --- | --- |
| Mock | PB2-Wild type | PB2-E362G | PB2-D441N |
| 0 | 16 | 16 | 16 |
| 0 | 8 | 32 | 8 |
| 0 | 8 | 16 | 16 |

Supplementary Table 6 Viral loads in the lungs of BALB/c mice infected with recombinant H5N1 viruses

| **Viral TCID₅₀ in lung** | |
| --- | --- |
| PB2-M631L | PB2-362G+441N+631L |
| 1 | 3 |
| 1.5 | 3.5 |
| 1 | 3 |

CT-qPCR

| vRNA:M1 | | | | | mRNA:M1 | | | | |
| --- | --- | --- | --- | --- | --- | --- | --- | --- | --- |
| **WT** | **E362G** | **D441N** | **M631L** | **E362G+D441N+M631L** | **WT** | **E362G** | **D441N** | **M631L** | **E362G+D441N+M631L** |
| 17.77 | 16.512 | 17.16 | 16.062 | 15.207 | 17.77 | 16.512 | 17.16 | 16.06 | 15.429 |
| 16.785 | 16.566 | 17.301 | 16.4004 | 15.52 | 16.785 | 16.566 | 17.301 | 16.4 | 15.52 |
| 17.285 | 16.926 | 17.137 | 15.963 | 15.559 | 17.285 | 16.926 | 17.137 | 15.96 | 15.559 |
| vRNA:NP |  |  |  |  | mRNA:NP |  |  |  |  |
| **WT** | **E362G** | **D441N** | **M631L** | **E362G+D441N+M631L** | **WT** | **E362G** | **D441N** | **M631L** | **E362G+D441N+M631L** |
| 16.559 | 17.536 | 16.48 | 15.809 | 14.832 | 16.559 | 17.536 | 16.48 | 15.809 | 14.832 |
| 16.449 | 17.34 | 16.582 | 15.746 | 14.707 | 16.449 | 17.262 | 16.582 | 15.746 | 14.707 |
| 16.371 | 17.262 | 16.465 | 15.926 | 14.879 | 16.371 | 17.379 | 16.465 | 15.926 | 14.806 |
